# Supplementary figures and images for: Advancing patient care: Machine learning models for predicting grade 3+ toxicities in gynecologic cancer patients treated with HDR brachytherapy
Source: PLoS One. 2025 May 14;20(5):e0312208. doi: 10.1371/journal.pone.0312208 (PMC12077677; doi:10.1371/journal.pone.0312208)

**(A) Disease Free Survival**

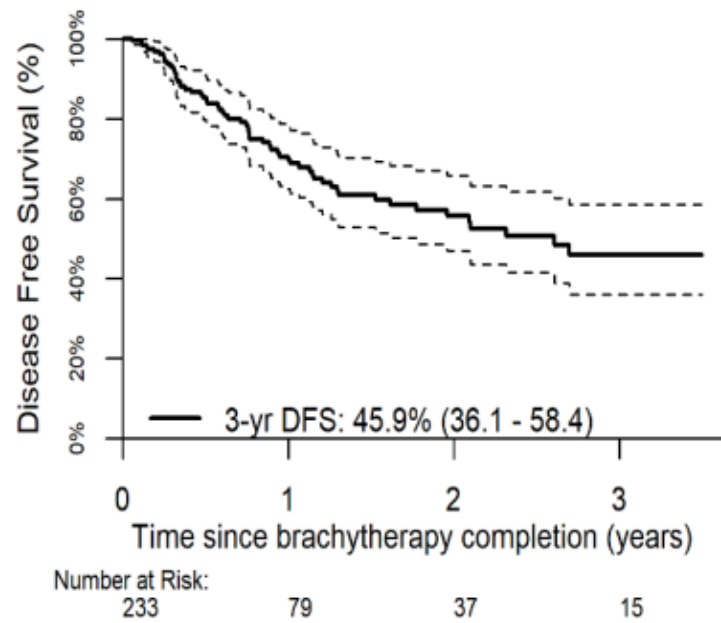

**(B) Local Control**

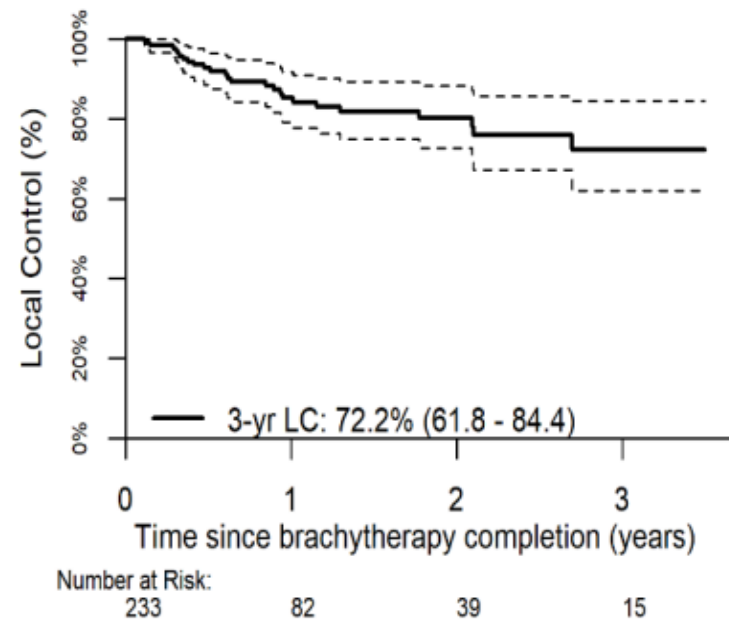

Supplement: S1 Fig — A) Disease Free Survival and B) Local control. (PDF) [file pone.0312208.s001.pdf]

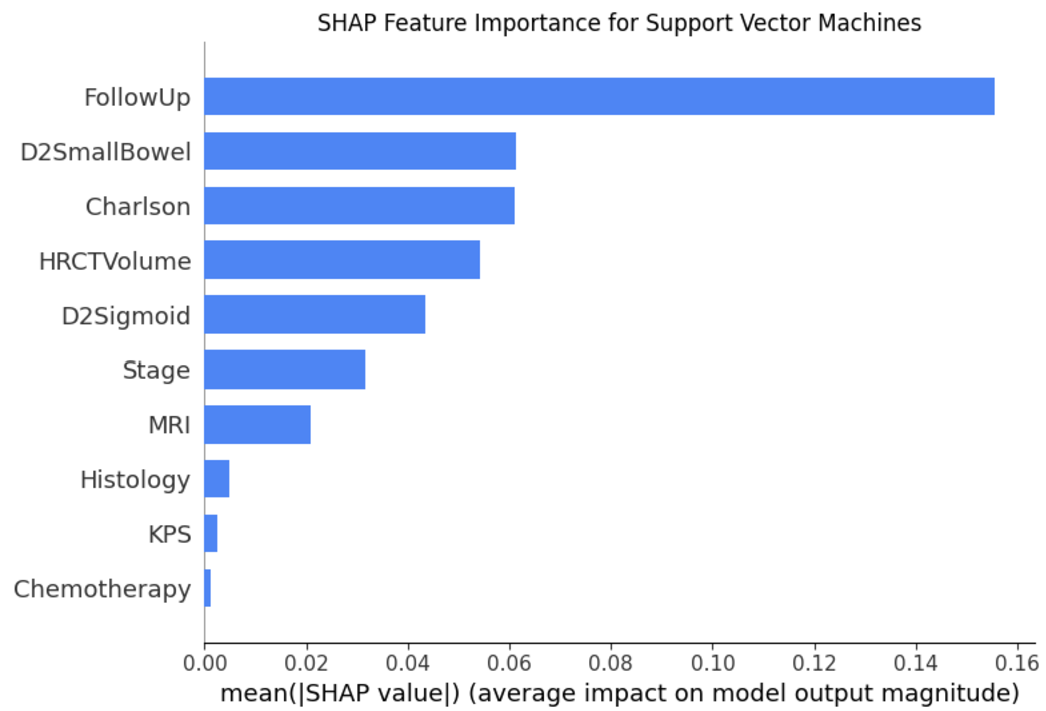

Supplement: S2 Fig — (PDF) [file pone.0312208.s002.pdf]

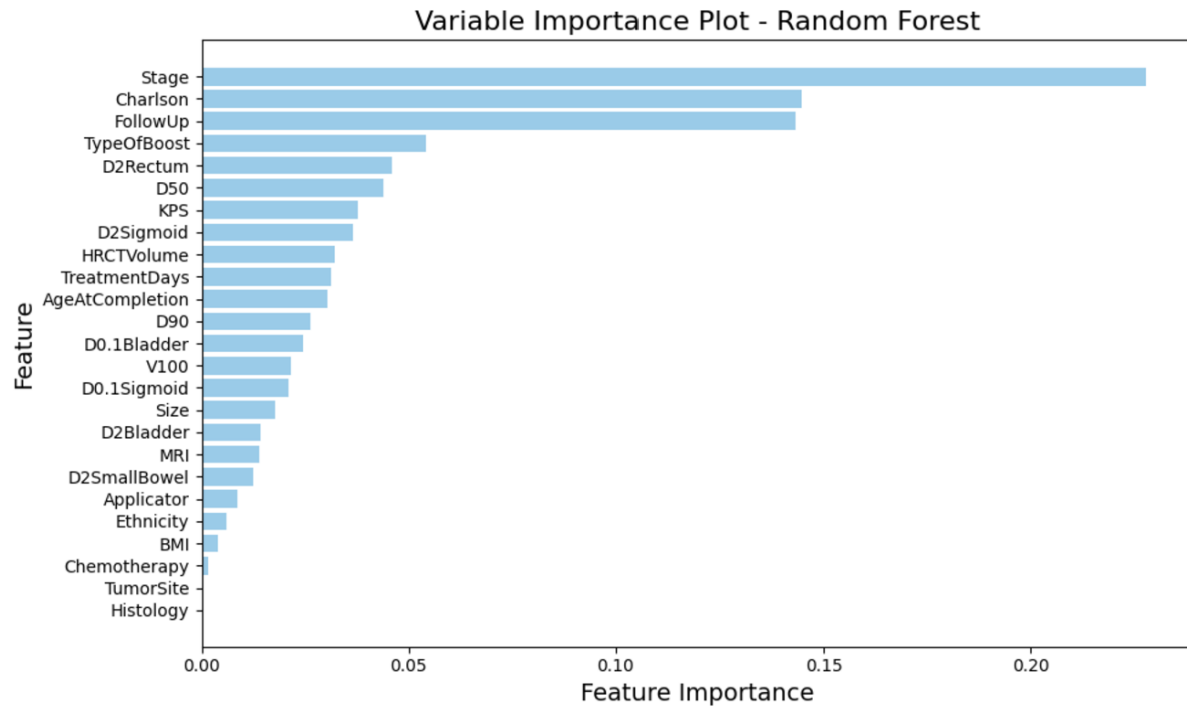

Supplement: S3 Fig — (PDF) [file pone.0312208.s003.pdf]

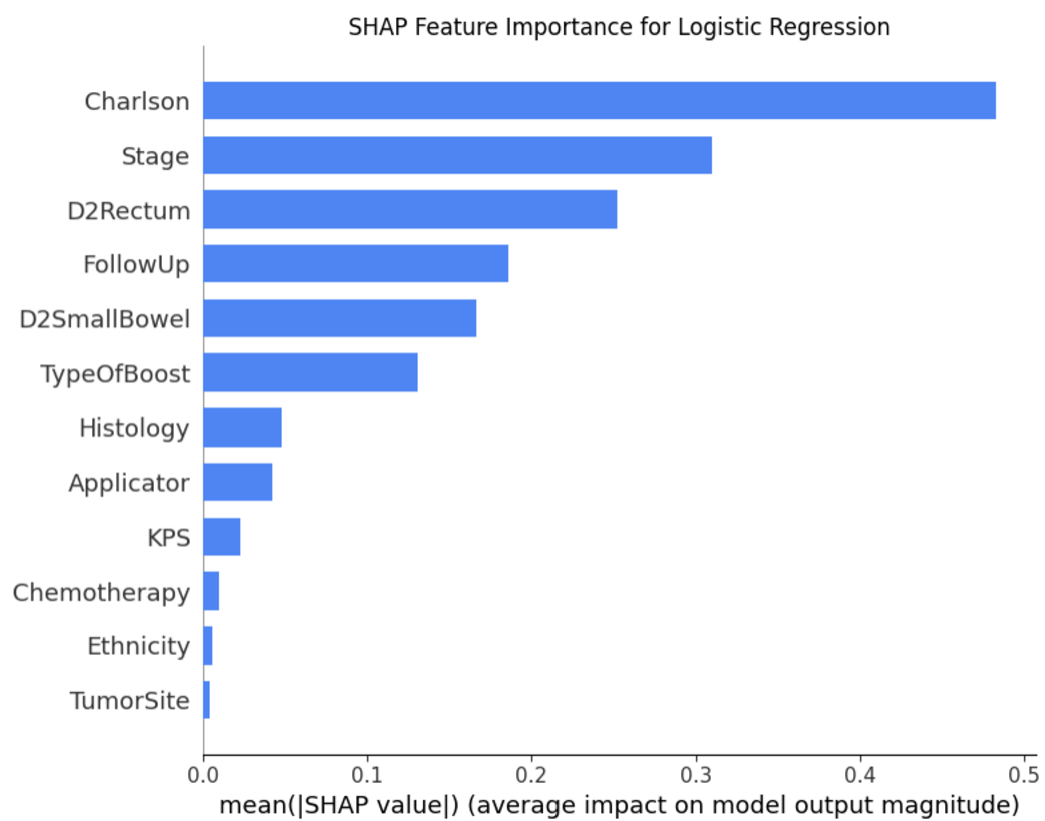

Supplement: S4 Fig — (PDF) [file pone.0312208.s004.pdf]
