# Supplementary material for: Advancing patient care: Machine learning models for predicting grade 3+ toxicities in gynecologic cancer patients treated with HDR brachytherapy
Source: PLoS One. 2025 May 14;20(5):e0312208. doi: 10.1371/journal.pone.0312208 (PMC12077677; doi:10.1371/journal.pone.0312208)
Supplement: S1 Table — (PDF) [file pone.0312208.s005.pdf]

Model	Hyperparameter Search Space
**SVM**	**kernel**: linear, rbf, poly, sigmoid<br>**Gamma**: Scale, Auto<br>**C**: [1 - 10]
**RF**	**min_samples_leaf**: [5 - 10]<br>**n_estimators**: [10, 15, 20, 25, 30, 35, 40, 45, 50, 100, 200]<br>**min_samples_split**: [5 - 10]<br>**max_features**: sqrt, log2
**LR**	**penalty**: l1, l2<br>**solver**:liblinear, lbfgs<br>**C**: [0.1, 0.3, 0.5, 0.7, 1]
**XGB**	**max_depth**: [1,2,3,4]<br>**n_estimators**: [75, 100, 125]<br>**learning_rate**: [0.01,0.1,0.2]<br>**subsample**: [0.5, 0.75, 1.0]<br>**colsample_bytree**: [0.5, 0.7, 1.0]<br>**gamma**: [0, 0.1, 0.2]
**MLP**<br>**2 Hidden Layers: (16,8)**	**learning_rate**: constant, invscaling, adaptive<br>**activation**: logistic, tanh, relu<br>**solver**: lbfgs, sgd<br>**learning_rate_init**: [0.001, 0.1, 0.15, 0.2, 0.3, 0.5, 1]<br>**alpha**: [0.0001, 0.0005, 0.001]<br>**batch_size**: [20, 32, 50]<br>**tol**: [0.00001, 0.0001, 0.0005]
**KNN**	**n_neighbors**: [1 - 10]
**GNB**	**var_smoothing**: [5e-08, 1e-09, 5e-09]
