# Supplementary material for: Advancing patient care: Machine learning models for predicting grade 3+ toxicities in gynecologic cancer patients treated with HDR brachytherapy
Source: PLoS One. 2025 May 14;20(5):e0312208. doi: 10.1371/journal.pone.0312208 (PMC12077677; doi:10.1371/journal.pone.0312208)
Supplement: S2 Table — The variable type and number of missing data points for each input is shown. (PDF) [file pone.0312208.s006.pdf]

N	Input				Output
	Feature Name	Feature Type	Missing Data Points	Number of Categories[a]	
1	Had Chemotherapy	Categorical	0	2	Develops toxicity grade 3 or higher
2	Body Mass Index (BMI)	Numerical	0	-	
3	Charlson Comorbidity Index (CCI)	Ordinal	0	4	
4	Karnofsky Performance Status (KPS)	Ordinal	1	3	
5	Ethnicity	Categorical	0	2	
6	Age at Completion [years]	Numerical	0	-	
7	EBRT Type of Boost	Categorical	0	3	
8	Treatment Days	Numerical	26	-	
9	Type of Applicator	Categorical	0	2	
10	MRI	Categorical	0	2	
11	V100 [cc]	Numerical	5	-	
12	D50 [Gy]	Numerical	6	-	
13	D90 [Gy]	Numerical	6	-	
14	D98 [Gy]	Numerical	6	-	
15	D0.1Bladder [Gy]	Numerical	8	-	
16	D1Bladder [Gy]	Numerical	8	-	
17	D2Bladder [Gy]	Numerical	8	-	Does not develop toxicity grade 3 or higher
18	D0.1SmallBowel [Gy]	Numerical	10	-	

19	D1SmallBowel [Gy]	Numerical	10	-	
20	D2SmallBowel [Gy]	Numerical	10	-	
21	D0.1Sigmoid [Gy]	Numerical	7	-	
22	D1Sigmoid [Gy]	Numerical	7	-	
23	D2Sigmoid [Gy]	Numerical	7	-	
24	D0.1Rectum [Gy]	Numerical	6	-	
25	D1Rectum [Gy]	Numerical	6	-	
26	D2Rectum [Gy]	Numerical	6	-	
27	Tumor Site	Categorical	0	3	
28	Cancer Stage	Ordinal	2	4	
29	Histology	Categorical	0	2	
30	Maximum Tumor Length [cm]	Numerical	39	-	
31	HRCTV Volume [cc]	Numerical	0	-	
32	Follow Up [months]	Numerical	0	-	

[a]The number of categories is also shown for categorical and ordinal variable types.
