# Supplementary material for: Advancing patient care: Machine learning models for predicting grade 3+ toxicities in gynecologic cancer patients treated with HDR brachytherapy
Source: PLoS One. 2025 May 14;20(5):e0312208. doi: 10.1371/journal.pone.0312208 (PMC12077677; doi:10.1371/journal.pone.0312208)
Supplement: Attachment — Submitted filename: Response to Reviewers.docx [file pone.0312208.s009.docx]

Academic Editor

PLOS ONE

Dear Dr. Li Yang;

Title: Advancing Patient Care: Machine Learning Models for Predicting Grade 3+ Toxicities in Gynecologic Cancer Patients treated with HDR Brachytherapy

Thank you for your valuable suggestions and remarks from the editor and reviewers. After incorporating your feedback our manuscript has greatly improved. Please find below the point-by-point responses to your comments. We hope that the present version of the manuscript will be acceptable for publication, and we look forward to your feedback.

**Referee #1's Comments**

This manuscript utilizes various machine learning methods to identify clinical features predicting toxicities from HDR brachytherapy in gynecological cancers. The manuscript demonstrates a high level of completeness in its application of machine learning, making it a valuable reference.

1. **Background information on toxicity from brachytherapy in gynecological cancers (definitions, incidence, causes, outcomes, etc.).**

**Response:** Thank you very much for your suggestions, background information on toxicities is extremely relevant to our work, and we have added the following paragraph:

“Possible side effects for patients who have undergone radiation treatment for gynecological cancers with a brachytherapy component include gastrointestinal (GI), genitourinary (GU), and vaginal (VAG) toxicities. According to the Common Terminology Criteria for Adverse Events (CTCAE) version 5.0, these toxicities are graded from 1 (mild) to 5 (death) based on their severity and impact on daily activities. Common GI toxicities include diarrhea, proctitis, and rectal bleeding. GU toxicities often present as urinary frequency, incontinence, or cystitis. Vaginal toxicities such as stenosis, mucositis, and fistulas may also occur. Follow-up data from the EMBRACE-I trial reported that at three years, the incidence of severe (grade 3 or higher) GI, GU, and vaginal toxicities were 7% (5.6–8.8%), 6.1% (4.8–7.7%), and 3.6% (2.7–5.0%), respectively. The overall incidence of adverse events, including non-GI, GU, and VAG toxicities, was 21.5% at three years and 26.6% at five years.”

Vittrup AS, Kirchheiner K, Pötter R, et al. Overall severe morbidity after chemo-radiation therapy and magnetic resonance imaging-guided adaptive brachytherapy in locally advanced cervical cancer: results from the EMBRACE-I study. *Int J Radiat Oncol Biol Phys*. 2023;116(4):807-24. doi:[10.1016/j.ijrobp.2023.01.002](https://doi.org/10.1016/j.ijrobp.2023.01.002)

2. **In Abstract: An overview of current research advancements in predicting toxicity from brachytherapy.**

**Response:** Thank you very much for your feedback. We have included a brief overview of current research in the Background section of the abstract as requested. The full paragraph now reads:

“Gynecological cancers are among the most prevalent cancers in women worldwide. Brachytherapy, often used as a boost to external beam radiotherapy, is integral to treatment. Advances in computation, algorithms, and data availability have popularized machine learning. Recent studies have applied machine learning models such as logistic regression, support vector machines, and deep learning networks to predict specific toxicities in patients who have undergone brachytherapy.”

3. **Materials and Methods: Although the authors included a substantial number of cases from a single center over a long period, using only 233 samples for training (with 75% allocated for training) is relatively small. The limitations of sample size should be addressed in the discussion.**

**Response:** Thank you for raising this point about sample size. While this sample size is larger than other studies of similar nature, we agree that the sample size is still a limitation. We have addressed this very important point by modifying the following paragraph:

“The primary limitation of this study is the relatively small sample size. Although our dataset of 233 data points is larger than those used in previous studies, only 75% of this data was utilized for training, which may influence the model’s accuracy and introduce uncertainty. Research has shown that smaller datasets are more prone to overfitting and can be influenced by random variability in the data [64]. The authors plan on elaborating multi-institutional studies to address sample size limitations in the future. Additionally, only the dosimetric, treatment, and tumor variables were considered in this study, but not any additional features such as dose maps…”

Rajput D, Wang WJ, Chen CC. Evaluation of a decided sample size in machine learning applications. *BMC Bioinformatics*. 2023;24(1):48. doi:10.1186/s12859-023-05156-9

4. **An explanation of the missing data situation is necessary, along with the rationale for choosing KNN among various imputation methods (advantages and disadvantages).**

**Response:** Thank you very much for your comment. We have adjusted the following paragraph to better justify our choice of the KNN imputer:

“Imputation of missing feature values was done via the K-nearest neighbors (KNN) algorithm due to its simplicity, low computational cost and its better overall performance over other data imputation methods such as MLP or mean/mode imputation. This approach aligns with the recommendations of Garcia-Laencina et al [24]. The missing data points primarily result from unobtainable patient records or measurements not being taken, suggesting the missingness is Missing at Random (MAR). As it matches an individual with missing data to a similar patient based on the observed data, KNN imputation is appropriate for MAR imputation when there is only a moderate amount of missingness (15%-30% missing), as in our setting. The advantages of KNN may be less pronounced with lower levels of missing data, as suggested by Beretta et al [25].”

García-Laencina PJ, Sancho-Gómez JL, Figueiras-Vidal AR. Pattern classification with missing data: a review. *Neural Comput Appl*. 2010;19:263-82. doi:10.1007/s00521-009-0295-6

Beretta L, Santaniello A. Nearest neighbor imputation algorithms: a critical evaluation. *BMC Med Inform Decis Mak*. 2016;16:197-208. doi: 10.1186/s12911-016-0318-z

5. **Reviewer Comment 5: Although the manuscript selects features such as Chemotherapy, Charlson Comorbidity Index, KPS, D2cc - Small Bowel, Stage, Histology, and Follow-Up Time, it does not clarify how these indicators are balanced and utilized in predicting toxicity.**

**Response:** Thank you very much for providing your valuable feedback. We are now including a detailed analysis of the most relevant features for the top 3 models by utilizing a SHAP analysis (For Support Vector Machines and Logistic Regression) and a variable importance plot for Random Forest (As suggested also by reviewer #3).

1) The following was added in the Materials and Methods section, at the end of the Feature Selection sub-section: “... A SHAP analysis [45] or variable importance plot (For RF) was done for the top 3 models to study the relevance of the final features that were included. “
2) Under Discussion, the following sentence was deleted: “One should keep in mind that the practical importance of each feature within an ML algorithm may vary and their relevance to the outcome should not be inferred solely based on their inclusion in the model.”
3) The following sentences were added, where the previous sentence was deleted: “Our top-performing model selected 10 features, with Length of Follow-Up, D2cc to the Small Bowel, Charlson Comorbidity Index, HR-CTV volume, and D2cc to the sigmoid being the most relevant (S2 Fig), agreeing with the discussed literature and the statistical analysis. Notably, Length of Follow-Up and Charlson Comorbidity Index were also significant predictors in our second and third best-performing models (S3 and S4 Fig). Higher D2cc doses to the small bowel and sigmoid indicate greater radiation exposure, increasing the risk of severe toxicity, while a higher Charlson Comorbidity Index reflects poorer overall health and greater susceptibility to adverse effects. Larger HR-CTV volumes may necessitate more aggressive treatment, heightening toxicity risk, and Length of Follow-Up is crucial for capturing late-onset toxicities.”

Lundberg S, Lee S. A unified approach to interpreting model predictions. *Adv Neural Inf Process Syst*. 2017;30:4765-74.

6. **Could constructing a multi-feature combined model provide more precise predictions? If further analysis is not feasible, please acknowledge the limitations in the discussion.**

**Response:** Thank you for this suggestion. A Multi-Feature Combined model analysis is an interesting approach that we could use to train new models, the authors will explore this method in future studies. We have modified the following paragraph in the limitations section to acknowledge this:

“Finally, more computationally expensive options should be explored. An in-depth analysis of the importance of each hyperparameter could be done in the future using packages such as the Optuna library [66]; additional ensemble models such as XGBoost could be trained and assessed; a combination of different models could also be explored, or a combination of features with a Multi-Feature Combined model as suggested by Yang et al [67].”

Yang Z, Wang C, Wang Y, Lafata KJ, Zhang H, Ackerson BG, Kelsey C, Tong B, Yin FF. Development of a multi-feature-combined model: proof-of-concept with application to local failure prediction of post-SBRT or surgery early-stage NSCLC patients. *Front Oncol*. 2023;13:1185771. doi:10.3389/fonc.2023.1185771

7. **While the manuscript offers an extensive discussion on the technical aspects of machine learning, the clinical application section is notably lacking.**

**Response:** Thank you very much for your comments. The clinical application section is extremely important and was indeed missing from the discussion section originally. To remediate this issue, the following paragraph was added.

“From a clinical application standpoint, ML models can significantly enhance patient care by identifying individuals who are at higher risk of developing severe toxicities. Clinicians can use this predictive information to modify the treatment plan to reduce the risk of severe toxicities, tailor patient counseling, set appropriate expectations, and implement proactive monitoring strategies. For example, patients identified as high-risk may benefit from more frequent follow-up appointments, early interventions for symptom management, or a change in the prescription dose. Additionally, the model can aid in shared decision-making by involving patients in discussions about the potential risks and benefits of their treatment plans. By stratifying patients based on their predicted toxicity risk, healthcare teams can allocate resources more efficiently and personalize supportive care measures to mitigate adverse effects. Thus, ML models can serve as a valuable tool for enhancing patient outcomes through individualized clinical management.”

**Referee #2's Comments**

1. **In Figure 1 the text is not readable.**

**Response**: Thank you very much for your comments. We have increased the font size of the text as suggested.

2. **The purpose section should be emphasized better.**

**Response:** Thank you very much for your suggestion. We have modified the purpose section and it now reads as follows:

This study aims to build and compare some of the more common binary classification machine learning models in the context of predicting if a patient will develop grade 3 or higher toxicities (Output: Yes/No) in gynecologic cancer patients treated with EBRT and brachytherapy. By accurately forecasting severe toxicities based on clinical, demographic, and treatment-related features, we aim to enhance personalized patient care by enabling clinicians to identify high-risk patients before treatment initiation. This predictive capability allows for proactive adjustments to treatment plans, and implementation of preventive measures regarding potential side effects. This study forms part of a larger research effort aimed at improving treatment outcomes and quality of life for patients by integrating machine learning models into the clinical decision-making process.

3. **Which software did you analyze with?**

**Response:** Thank you very much for noticing that we were missing the software we employed for the traditional statistical analysis. Now it is mentioned at the end of the paragraph of the statistical analysis and reads as follows:

“... This analysis was performed using R statistical software version 4.3.2. [22].”

R Core Team (2024). R: A language and environment for statistical computing. R Foundation for Statistical Computing, Vienna, Austria. Available at: https://www.R-project.org/. Accessed November 25, 2024.

A sentence was also added to the Data Preprocessing and now reads:

“The machine learning analysis was done using Python 3 and Jupyter Notebook (IPython kernel). Various code libraries (collections of pre-written functions and classes), including Scikit-learn v1.3.2 [23]”

4. **Can you give information about analysis times? Also provide information about the computer on which you performed the analysis.**

**Response:** Thank you very much for pointing out that we were missing the time it took for the models to train. We have now included both the times, and information about the computer that was used to obtain these. This data is presented in the results section and reads as follows:

“The total runtime for the training of Logistic Regression, Random Forest, K-Nearest Neighbors, Support Vector Machines, Gaussian Naive Bayes, and Multi-Layer Perceptron was 0.38, 9.96, 0.62, 0.74, 0.14, and 110 minutes, respectively. The Python script was executed on a computer equipped with an 14th Generation Intel(R) Core(TM) i9-14900HX 2.20 GHz processor (E-cores up to 4.10 GHz P-cores up to 5.80 GHz), 32 GB of DDR5-5600 MHz (SODIMM) RAM, and a 64-bit Windows 11 Pro operating system.

5. **Write the limitations of the methods.**

**Response:** Thank you for raising this observation. The authors have expanded the limitations of methods in the following ways:

1. We have added a section discussing MLP and KNN overfitting, and some potential solutions:

“Furthermore, developing methods to address overfitting could be beneficial, particularly for MLP and KNN. For KNN, different alternatives for the classifier could be explored, such as weighted-KNN, Radius-based Nearest Neighbor, or changing to a different distance metric other than Euclidean. For MLP, a much more defined hyperparameter space will be addressed in future studies. Several overfitting solutions have been outlined by Ying [65], such as Early-Stopping, L1 and L2 regularization parameters, etc.”

2. We have also added a section discussing the hyperparameter search space, their relative importance, alternative model options we can explore, and the combination of features:

Finally, more computationally expensive options should be explored. An in-depth analysis of the importance of each hyperparameter could be done in the future using packages such as the Optuna library [66]; additional ensemble models such as XGBoost could be trained and assessed; a combination of different models could also be explored, or a combination of features with a Multi-Feature Combined model as suggested by Yang et al [67].

6. **Add the limitations of the study and your suggestions to the results.**

**Response:** Thank you for your feedback. We have expanded on our previous limitation section in the following ways:

1. We have expanded upon our sample size limitation by adding:

“The primary limitation of this study is the relatively small sample size. Although our dataset of 233 data points is larger than those used in previous studies, only 75% of this data was utilized for training, which may influence the model’s accuracy and introduce uncertainty. Research has shown that smaller datasets are more prone to overfitting and can be influenced by random variability in the data [64]. The authors plan on elaborating multi-institutional studies to address sample size limitations in the future.”

**Referee #3's Comments**

In this work, the authors used data from a retrospective study of 233 patients to train statistical and machine learning models to predict grade 3 or higher toxicities in gynecological cancer patients treated with brachytherapy. Overall, I thought the paper was well written, easily readable, and would be interesting to applied researchers in the fields of oncology and gynecology. My experience only allows me to comment on the validity of this work with respect to the data modeling strategies utilized. In my opinion, the methods are well chosen but could use further clarification. Specified points for potential improvement are below.

1. **Line 77: A useful citation for this line might be Breiman’s 2001 paper on “Statistical Modeling: The Two Cultures”.**

**Response:** Thank you very much for your suggestion. This citation is indeed very relevant and interesting; we have added it to the manuscript.

Breiman L. Statistical modeling: The two cultures (with comments and a rejoinder by the author). Statistical science. 2001;16(3):199-231. doi:10.1214/ss/1009213726

2. **Beyond the sample size, either in the introduction or results section, it would be beneficial to report the total number of features, the number of features that are categorical, ordinal, or continuous, and if any features had a high level of missingness.**

**Response:** Thank you very much for your comment. Indeed, it is a good idea to input into the main text the details of S2 Table to clarify the total number of features, their variable types, and their level of missingness. The first paragraph of the results section now reads:

“The dataset included demographic and clinical data for n = 233 patients, of which n = 56 (24%) had a grade 3 or higher toxicity. It comprised 32 features: 3 ordinal, 7 categorical, and 22 numerical variables. The highest missing data rates were observed for the total number of treatment days (11%) and maximum tumor length (16.7%), while all other features had missingness rates of 4.3% or less”

3. **Line 158: Please explain further why Target Encoding was chosen as opposed to using something like one-hot encoding.**

**Response:** Thank you very much for your comment. We have further elaborated on our decision to use Target Encoder instead of One Hot Encoding by adding the following:

“For categorical and ordinal variables, both Target Encoding and One Hot Encoding were initially considered. Due to our sample size, the former was ultimately favored due to it not increasing the number of features, given how an increased features-to-samples ratio has been associated with overfitting the data [30].”

As seen by Vabalas et al. : “the higher the ratio of features to sample size the more likely that an ML model will fit the noise in the data instead of underlying pattern.”

Vabalas A, Gowen E, Poliakoff E, Casson AJ. Machine learning algorithm validation with a limited sample size. PloS one. 2019;14(11):e0224365. doi:10.1371/journal.pone.0224365

4. **In Fig 1, it is shown that the Standard Scaler operation is applied to the training and testing data. Is the same standard scaling that was applied to the training set the same as the test set? If so, it could be helpful to clarify this. If not, this is a potential issue since the models were fitted on a different scale.**

**Response:** Thank you very much for your feedback. Indeed, the same standard scaling that was applied to the training set is the same as the one applied to the testing set. The following two sentences help show this:

1) Under Data Pre-Processing: “They were fitted using the training data only, and their algorithms applied to both the training and testing data [21].”
2) Figure 1 Description: “(iv) a *StandardScaler* is fitted and applied to the training data ensuring they are on a comparable scale. (C) Data preprocessing (Testing Set): The preprocessing objects fitted to the training set are subsequently applied to the testing set:”

As suggested by the reviewer, this is an important step that needs to be emphasised. In order to do so, we have modified the picture to show what operations were done on each data set for the pre-processing (Fit_Transform vs Transform). It now shows:

5. **Line 209 – Are the models listed in parentheses the only ones that SFS was applied to? Might want to clarify this more**

**Response:** Thank you very much for your comments. The models for which SFS was applied are now mentioned directly in the Sequential Feature Selection section. It now reads:

“... after experimentation, Sequential Forward Selection was chosen and applied during the training of LR, SVM, KNN and GNB due to faster computation times and better performance metrics.”

6. **Line 300 – Are there any conjectures that could be included as to why these models over-fit but the others don’t?**

**Response:** Thank you for the observation. We have included our main speculation as to why these two models have overfitted our data:

“To mitigate this phenomenon, the use of a withheld testing data set is required to assess the degree of overfitting and the performance of the model [54]. The authors recommend that the training and withheld data testing scores should always be reported to provide a comprehensive understanding of a model’s performance. A clear illustration of overfitting can be appreciated in Table 3 for the MLP and KNN models, where they achieved impressive training F1 scores of 1.00 and 0.72 respectively; contrasting sharply with their testing scores of 0.39 and 0.32. These scores show that these 2 models are not generalizable for predicting new similar data points. A likely explanation for these two models' performance would be their hyperparameter sensitivity. In the case of MLP, there are a large number of hyperparameters and possibilities for neuron activation; on the other hand, KNN relies on a highly accurate selection of *k-neighbors* to make a reliable classification [62]. Each model merits a robust and thorough search space to cover sufficient model possibilities, which the authors plan on exploring with more advanced hyperparameter tuning packages. ”

Zhang Z. Introduction to machine learning: k-nearest neighbors. *Ann Transl Med*. 2016;4(11). doi:10.21037/atm.2016.03.37

The following was also added regarding KNN:

“… For KNN, different alternatives for the classifier could be explored, such as weighted-KNN, Radius-based Nearest Neighbor, or changing to a different distance metric other than Euclidean. For MLP, a much more defined hyperparameter space will be addressed in future studies. Several overfitting solutions have been outlined by Ying [65], such as Early-Stopping, L1 and L2 regularization parameters, etc.”

7. **Table 3 – Maybe bold values to show best scores.**

**Response:** Thank you for your suggestion. We have bolded the best performance metrics obtained by the testing data.

8. **Line 389 – In the conclusion, regularization methods for MLP and KNN models could be mentioned to fix the issue of overfitting.**

**Response:** Thank you for this suggestion. We have addressed some potential ways to solve overfitting for MLP and KNN in the following paragraph:

Furthermore, developing methods to address overfitting could be beneficial, particularly for MLP and KNN. For KNN, different alternatives for the classifier could be explored, such as weighted-KNN, Radius-based Nearest Neighbor, or changing to a different distance metric other than Euclidean. For MLP, a much more defined hyperparameter space will be addressed in future studies. Several overfitting solutions have been outlined by Ying [65], such as Early-Stopping, L1 and L2 regularization parameters, etc.

Ying X. An overview of overfitting and its solutions. *Journal of physics: Conference series*. 2019;1168:022022. doi:10.1088/1742-6596/1168/2/022022

9. **S1 Table – RF predictors become more stable as the number of estimators increases. Why was 50 chosen as the largest number of trees as opposed to something on the order of 100s?**

**Response:** Thank you for your comment on RF predictors. The hyperparameter tuning was performed again by adding 100 and 200 trees to the hyperparameter search space; the best number of estimators remained at 15. We have updated S1 Table accordingly to include these new search parameters.

10. **Some features have a fair amount of missingness (17% for maximum tumor length and 11% for Treatment Days). It would be worthwhile to further clarify why the specific imputation method was chosen. Usually, the method chosen to deal with missing data is based on the expected type of missingness (e.g. missing completely at random vs missing at random). Either way, the variables with large degrees of missingness don’t appear to be included in the top 3 final models so it doesn’t seem like the imputation method should have a large impact.**

**Response:** Thank you for the insightful comment. The authors have expanded on the choice of KNN Imputer by elaborating on the paragraph below:

“Imputation of missing feature values was done via the K-nearest neighbors (KNN) algorithm due to its simplicity, low computational cost and its better overall performance over other data imputation methods such as MLP or mean/mode imputation. This approach aligns with the recommendations of Garcia-Laencina et al [24]. The missing data points primarily result from unobtainable patient records or measurements not being taken, suggesting the missingness is Missing at Random (MAR). As it matches an individual with missing data to a similar patient based on the observed data, KNN imputation is appropriate for MAR imputation when there is only a moderate amount of missingness (15%-30% missing), as in our setting. The advantages of KNN may be less pronounced with lower levels of missing data, as suggested by Beretta et al [25].”

García-Laencina PJ, Sancho-Gómez JL, Figueiras-Vidal AR. Pattern classification with missing data: a review. *Neural Comput Appl*. 2010;19:263-82. doi:10.1007/s00521-009-0295-6

Beretta L, Santaniello A. Nearest neighbor imputation algorithms: a critical evaluation. *BMC Med Inform Decis Mak*. 2016;16:197-208. doi: 10.1186/s12911-016-0318-z

11. **The random forest model has all the features included. An interesting result to report could be a VarImp plot or some other variable importance metric to see if the important features align with the other models or the univariate analyses.**

**Response:** Thank you very much for your suggestion. We agree it would be relevant to the paper to explain the importance of the variables in the final models; in order to provide this information, we have followed your recommendation and done a VarImp plot for the Random Forest Model, and a SHAP analysis for SVM and LR (To analyze all top 3 models) [Available for visualization in S2, S3, and S4 Fig]. We have added the following paragraph to the Discussion section:

“Our top-performing model selected 10 features, with Length of Follow-Up, D2cc to the Small Bowel, Charlson Comorbidity Index, HR-CTV volume, and D2cc to the sigmoid being the most relevant (S2 Fig), agreeing with the discussed literature and the statistical analysis. Notably, Length of Follow-Up and Charlson Comorbidity Index were also significant predictors in our second and third best-performing models (S3 and S4 Fig). Higher D2cc doses to the small bowel and sigmoid indicate greater radiation exposure, increasing the risk of severe toxicity, while a higher Charlson Comorbidity Index reflects poorer overall health and greater susceptibility to adverse effects. Larger HR-CTV volumes may necessitate more aggressive treatment, heightening toxicity risk, and Length of Follow-Up is crucial for capturing late-onset toxicities”

12. **To further strengthen the argument that the SVM, RF, and LR tend to perform best, it would be interesting to see a subset of the analysis repeated on resampled train/test splits of the original data. However, it is understandable if this would be too computationally expensive to include.**

**Response:** The authors agree that using multiple subsets or bootstrapping would be ideal to evaluate the stability and robustness of the model. We have calculated the confidence intervals by doing bootstrapping instead of applying the CLT, and updated Table 4 accordingly. The methodology now reads as follows:

“Confidence intervals of 95% were calculated using bootstrap resampling with replacement over 1000 iterations. In each iteration, the model was retrained using balanced data, and metrics were recalculated on the unbalanced training and testing datasets. The standard deviation of the metric values across iterations was used to estimate the confidence interval under the assumption of a normal distribution [36].”
