# Supplementary material for: Advancing patient care: Machine learning models for predicting grade 3+ toxicities in gynecologic cancer patients treated with HDR brachytherapy
Source: PLoS One. 2025 May 14;20(5):e0312208. doi: 10.1371/journal.pone.0312208 (PMC12077677; doi:10.1371/journal.pone.0312208)
Supplement: Attachment — Submitted filename: comments.docx [file pone.0312208.s008.docx]

The article was reviewed under the title "Advancing patient care: Machine learning models for predicting grade 3+ toxicities in gynecologic cancer patients treated with HDR brachytherapy".

Overall, the focus of the study is good. However, I would like to offer several recommendations that authors may find useful in the process of revising their manuscript:

1- Given the importance of the research topic, it is necessary to examine previous studies in more detail.
2- Accordingly, a comparison between the results obtained and other research can be described.
3- Considering the recent research conducted on gynecological diseases based on deep learning methods, is there a specific reason for choosing classical machine learning methods?
4- Some of the sources used are old, and given the more recent research, it is better to use newer sources for reference.
5- Given the need for large training data for machine learning models, is there a reason to use small amounts of data?
