# Supplementary material for: Advancing patient care: Machine learning models for predicting grade 3+ toxicities in gynecologic cancer patients treated with HDR brachytherapy
Source: PLoS One. 2025 May 14;20(5):e0312208. doi: 10.1371/journal.pone.0312208 (PMC12077677; doi:10.1371/journal.pone.0312208)
Supplement: Attachment — Submitted filename: Response to Reviewers - Round 2.docx [file pone.0312208.s011.docx]

Academic Editor

PLOS ONE

Dear Dr. Li Yang;

Title: Advancing Patient Care: Machine Learning Models for Predicting Grade 3+ Toxicities in Gynecologic Cancer Patients treated with HDR Brachytherapy

Thank you for your valuable suggestions and remarks from the editor and reviewers in this second round. We are very pleased to hear we addressed all the initial reviewer comments. After incorporating this new feedback, we feel our manuscript has greatly improved. Please find below the point-by-point responses to your comments. We hope that the present version of the manuscript will be acceptable for publication, and we look forward to hearing from you.

**Referee #5's Comments**

Overall, the focus of the study is good. However, I would like to offer several recommendations that authors may find useful in the process of revising their manuscript:

1. **Given the importance of the research topic, it is necessary to examine previous studies in more detail.**

**Response:** Thank you for your suggestion. We have added the following sentences relating to other studies where ML has been applied for cervical cancers treated with High Dose Rate Brachytherapy.

“In treatment of cervical cancer with HDR brachytherapy, ML has proven to be applicable to several aspects. For instance, tree-based models have been found to provide accurate classification performance for optimal applicator selection [13], and deep-learning methods show promise in improvement of treatment planning, such as segmentation, reconstruction, plan optimization, dose calculation, and other aspects related to treatment outcomes [14].”

2. **Accordingly, a comparison between the results obtained and other research can be described.**

Response: Thank you very much for your comment. We have added some sentences comparing our results to other relevant studies. However, it is important to note that our study is the first to specifically develop ML models for predicting Grade 3 or higher toxicities, which limits the possibility of a direct comparison. The revised discussion now reads:

“Regarding ML in brachytherapy toxicity prediction, Tian et al. developed a model for predicting fistula formation, reporting a recall of 97.1% and AUC of 0.904 utilizing the SMOTE algorithm and a SVM model with a radial basis kernel function on a database that included 35 patients with 7 positive cases; the limitation of this study lies in the small dataset, no withheld test dataset, high risk of model overfitting, and only using one model in their study [58]. For prediction of rectal toxicities, Chen et al. and Zhen et al. predicted grade 2 or higher rectal toxicity by using SVM and convolutional neural networks, respectively, with scores of (cross-validation estimated) recall and AUC of 0.85 and 0.91 for the former and 0.75 and 0.89 for the latter. Their work includes the addition of dose map features for the training of the model; both of these studies were done with a database of 42 patients with 12 positive cases of patients that developed toxicities [59-60]. Additionally, there has been work by Lucia et al. who developed Normal Tissue Complication Probability (NTCP) models for acute and late gastrointestinal, genitourinary, and vaginal toxicities using a database of 102 patients that included radiomic features, but only for a logistic regression model, which obtained balanced accuracy scores between 63.99 and 78.41 [61]. Cheon et al. considered deep learning models for predicting late bladder toxicities which outperformed its multivariable logistic regression counterpart [62], with data of 281 patients which achieved an F1 score of 0.76. In contrast to earlier investigations, our study presents the largest patient dataset used for predicting grade 3 or higher toxicities. Similar to these studies, we employ data-balancing algorithms to promote stability in the model training stage. Our methodology incorporates feature selection for all models except for MLP and RF. Specifically, we leverage the Sequential Feature Selection Algorithm to promote parsimony within the model fit. This aligns with the methodologies employed in previous reports.

Regarding the scoring metrics, our study showed that the support vector machine was the best model for predicting grade 3 toxicities, obtaining a training F1 score of 0.61, accuracy of 0.82, normMCC of 0.75, precision of 0.63, recall of 0.6, AUC-ROC of 0.87, and AUC-PR of 0.6; whereas for that same model, the test data obtained an F1 score of 0.63, accuracy of 0.80, normMCC of 0.75, precision of 0.56, recall of 0.71, AUC-ROC of 0.78, and AUC-PR of 0.65. In the withheld test data, out of all the patients that had a toxicity (n = 14), 71% were correctly predicted by the model (TP = 10); and out of all the predicted cases, 56% represented a true toxicity event and were not false positives (FP = 8). Given the high level of clinical uncertainty in whether patients will develop toxicities, this may be viewed as an adequate performance; while slightly lower, it remains comparable to similar studies, likely reflecting the increased difficulty associated with accurately predicting higher-grade (Grade ≥3) toxicities…”

3. **Line 209 – Considering the recent research conducted on gynecological diseases based on deep learning methods, is there a specific reason for choosing classical machine learning methods?**

**Response:** Thank you for raising this important point. The primary reason for selecting classical machine learning (ML) methods was the moderate size of our dataset (n=233), which poses practical challenges when training more complex deep learning models due to potential risks of overfitting. Classical ML algorithms such as logistic regression, random forest, and support vector machines generally perform reliably with datasets of this scale and also offer greater interpretability—both valuable considerations in clinical contexts. Nevertheless, we included a Multi-Layer Perceptron (MLP), a simpler form of neural network, in our analysis. The MLP’s performance did not substantially exceed that of the classical approaches, further supporting our methodological choice. We have explicitly clarified this rationale in the revised manuscript to reflect your feedback.

The paragraph now reads: “A total of 7 machine learning models were implemented and compared. The included models were the following: Logistic Regression (LR), Random Forest (RF), K-Nearest Neighbors, Support Vector Machines (SVM), Gaussian Naïve Bayes (GNB), Multi-Layer Perceptron Neural Network, and XGBoost (XGB). While there are many other ML classification algorithms in the literature, these seven choices represent the most commonly utilized algorithms for radiation outcome modeling [48]. The selection of these methods provided a range from simpler models (such as LR, KNN, and GNB) to more complex approaches (such as RF, SVM, MLP and XGBoost), addressing practical challenges related to dataset size and potential risks of overfitting.”

4. **Line 209 – Some of the sources used are old, and given the more recent research, it is better to use newer sources for reference.**

**Response:** Thank you very much for your comments. We agree having newer sources for reference is a good practice. We have updated references 3, 12, 18, 28, 35, 39 and 43.

5. **Line 209 – Given the need for large training data for machine learning models, is there a reason to use small amounts of data?**

**Response:** We appreciate the reviewer highlighting this important concern. The relatively small size of our dataset reflects the practical limitations in data availability typically encountered in radiation oncology, especially when focusing on a highly specific treatment modality like HDR brachytherapy for gynecologic cancers. Understanding this inherent limitation, we specifically selected a variety of ML methods, including simpler models proven effective with smaller datasets. Rather than a limitation per se, our dataset size underscores the importance of future multi-institutional collaborations, as pooling larger datasets could allow for training more complex models and potentially achieving improved predictive performance.

We modified the limitation paragraph and now the relevant sentence now reads as follows: “Research has shown that smaller datasets are more prone to overfitting and can be influenced by random variability in the data [67]. To address this, several models were evaluated, ranging from simpler methods to more complex machine learning algorithms.”

**Referee #6's Comments**

The manuscript introduces the results of a study that built and compared some of the more common binary classification machine learning models in the context of predicting if a patient is going to develop grade 3 or higher toxicities in gynecologic cancer patients treated with EBRT and brachytherapy. While the motivation and the relevance of the work is high, the approaches discussed not necessarily the most performant. The task itself is not a very complex one (i.e., binary classification) with a note that 3+ toxicities are not a large sample. Overall, the work has its merits through the thorough and systematic approach to evaluate and compare the results among the ML models.

1. **You mention on line 130 efficiency and reproducibility. I salute the code release but I would be careful on the "efficiency" statement. What does that mean in your case. Efficient implementations are typically linked to the underlying resources where the model are trained and used in inference. There we have different metrics for performance. Without values (training time, resources used), I would refrain to talk about efficiency.**

**Response:** Thank you very much for your comment. We agree that the word efficiency is ambiguous in this context and have modified the wording. The sentence now reads: “Various code libraries (collections of pre-written functions and classes), including Scikit-learn v1.3.2 [25], were used for their straightforward integration and reproducibility”

2. **On line 135 you mention imputation as a second step in your pipeline. Although you mention SOTA methods later, it is unclear why would you need it. Some of the models there also do well with little data, and given that the 3+ toxicity are relatively rare (see line 154 and 155) w.r.t. the total treated patients, this might need some motivation. I would argue you could do well enough with "good priors" and a simpler model.**

**Response:** We thank the reviewer for raising this important point, allowing us to clarify our approach. Our dataset included some missing clinical and dosimetric values, reflecting real-world scenarios where complete patient records are not always available.

Imputation was chosen specifically to maximize the utilization of our available dataset, given its relatively modest size (233 patients), as excluding incomplete cases would substantially reduce statistical power and potentially introduce selection biases. To address missingness robustly and effectively. We used K-Nearest Neighbors (KNN) imputation, which is a standard and appropriate method for clinical datasets. It provides slightly less classification error, and more importantly, much less computational cost than other methods such as Multilayer Perceptron (MLP) imputation, as highlighted by García-Laencina. Although simpler models or models leveraging strong priors might indeed perform adequately in certain contexts, our study aimed to rigorously compare multiple machine learning methods.

We have modified the paragraph in the Data Preprocessing sub-section to discuss this rationale; the following sentences have been added:

“Imputation was essential to allow consistent, unbiased comparison across multiple ML algorithms, including those which inherently cannot handle missing values, and to fully utilize our limited dataset without discarding cases.”

García-Laencina PJ, Sancho-Gómez JL, Figueiras-Vidal AR. Pattern classification with missing data: a review. Neural Comput Appl. 2010;19:263-82. doi:10.1007/s00521-009-0295-6

3. **I would suggest to also look at XGBoost as a candidate ML model for better performance as the out of the box SKLearn SVM and Random forest, multiple advantages event when considering efficiency (the optimized XGBoost model can run on a PC, tablet, even in the browser)**

**Response:** Thank you for suggesting looking at XGBoost as a potential model. We have trained and reported its results in the manuscript in all relevant sections; we now mention it initially in the Machine Learning Algorithms sub-section as follows:

“A total of 7 machine learning models were implemented and compared. The included models were the following: Logistic Regression (LR), Random Forest (RF), K-Nearest Neighbors, Support Vector Machines (SVM), Gaussian Naïve Bayes (GNB), Multi-Layer Perceptron Neural Network, and XGBoost (XGB).

The authors also value the feedback on cross-platform performance, additional discussion on model deployment can be found on comment number 7.

4. **The evaluation metrics are standard, I would move them in appendix, at least for the ML community it is redundant**

**Response:** Thank you very much for your feedback. We agree that the evaluation metrics are standard in the ML community, but this article is mainly oriented towards radiation oncology professionals. Due to the importance of selecting an appropriate metric for each case scenario, we believe it would be useful for the expected target group to keep them in the main manuscript body.

5. **The model choice is "a la textbook", rather than supported by intuitive insights on what, how, and why each model can provide for the task at hand**

**Response:** Thank you for your comment. We selected several of the most widely used machine learning algorithms in radiation outcome modeling and included additional simpler models, such as K-Nearest Neighbors, given our dataset's size. We have added a reference and clarified our rationale for model selection; the paragraph now reads:

“A total of 7 machine learning models were implemented and compared. The included models were the following: Logistic Regression (LR), Random Forest (RF), K-Nearest Neighbors, Support Vector Machines (SVM), Gaussian Naïve Bayes (GNB), Multi-Layer Perceptron Neural Network, and XGBoost (XGB). While there are many other ML classification algorithms in the literature, these seven choices represent the most commonly utilized algorithms for radiation outcome modeling [48]. The selection of these methods provided a range from simpler models (such as LR, KNN, and GNB) to more complex approaches (such as RF, SVM, MLP and XGB), addressing practical challenges related to dataset size and potential risks of overfitting.”

Luo Y, Chen S, Valdes G. Machine learning for radiation outcome modeling and prediction. *Med Phys*. 2020;47(5):e178-84. doi:[10.1002/mp.13570](https://doi.org/10.1002/mp.13570)

6. **The limitations (line 432) is not necessarily a limitation, is the task itself. Again, chosing model which can do well on small datasets is the key (here statistics and simpler models can do way better than MLP)**

**Response:** Thank you for your insightful comment. We agree that the dataset size is an inherent aspect of the task rather than strictly a limitation and selecting models well-suited for small datasets is indeed crucial. This rationale guided our decision to explore simpler models and more complex approaches, instead of just focusing on one model. Rather than a strict limitation, the smaller dataset reflects the realities of data availability in this field. We highlight it to underscore the importance of collaboration in radiation oncology: with larger pooled datasets, more complex ML models may prove more effective and yield stronger performance.

7. **How are you planning to use the results to close the loop for the benefit of the patients in therapy control/management and QoL? Any thoughts about deployment, turning this in an app for the physicians?**

**Response:** Thank you very much for your questions and feedback. We have included the relevant information in the second paragraph of the discussion, adding relevant information as suggested by the reviewer. It now reads:

“From a clinical application standpoint, ML models can significantly enhance patient care by identifying individuals who are at higher risk of developing severe toxicities. Clinicians can use this predictive information to modify the treatment plan to reduce the risk of severe toxicities, tailor patient counseling, set appropriate expectations, and implement proactive monitoring strategies. For example, patients identified as high-risk may benefit from more frequent follow-up appointments, early interventions for symptom management, or a change in the prescription dose. Additionally, the model can aid in shared decision-making by involving patients in discussions about the potential risks and benefits of their treatment plans. By stratifying patients based on their predicted toxicity risk, healthcare teams can allocate resources more efficiently and personalize supportive care measures to mitigate adverse effects and maintain or improve patients’ QoL. Thus, ML models can serve as a valuable tool for enhancing patient outcomes through individualized clinical management. Once validated, deployment of the best model into clinical workflows could be done with a decision-support application that provides real-time risk assessments during treatment planning. This tool could be implemented directly within the Eclipse environment via ESAPI scripting (C#), as a standalone browser-based application accessible to clinicians, or through alternative deployment methods adapted to the specific needs and infrastructure of each clinic.”

8. **Did you use physicians when considering priors one can inject in the ML models to make sure they extract the correct dependency between variables? -**

**Response:** Thank you very much for this question. It is indeed very relevant and important to highlight that the variables that were used to create this database were chosen with physician input. We have added the following sentence in the Methods section:

“A comprehensive diagram depicting the full machine learning workflow is provided in Fig 1, and S2 Table presents a summary of all the features explored by the ML models, along with their types. The initial features included in the database of study were selected through discussions with physicians and professionals directly involved in HDR treatments.

9. **Physics-informed ML or PhyML is a strong candidate to introduce and induce known (mechanistic priors) into the system and when complemented with simulation you can go beyond the statistical imputation of data**

**Response:** Thank you for your comment. We appreciate your suggestion regarding physics-informed machine learning (PhyML) to introduce mechanistic priors and reduce reliance on purely statistical imputation. While PhyML holds promise in many areas of radiation oncology, the clinical context of HDR brachytherapy poses unique challenges. Specifically, patient anatomy, applicator orientation, and organ motion can vary significantly, and the final isodose distribution often depends on physician or physicist manual adjustments in the treatment planning system (TPS). These factors make it difficult to apply universal physics-based or geometry-based relationships without extensive customization, as each insertion may involve a unique setup tailored to the patient’s individual needs.

Nevertheless, we acknowledge that future studies could integrate TG-43’s deterministic formula with standardized dwell-time patterns and applicator parameters to generate initial dose estimates. For instance, one could define typical dwell times for each channel in a given applicator, based on standard treatment durations, tumor volume, and proximity to OARs for a specified HDR source activity. TG-43 could then compute a preliminary isodose distribution from these averages. A machine-learning layer might subsequently refine or “correct” that baseline to account for real-world variations—such as physician plan adjustments or complex anatomical differences—in a more physics-informed manner. Determining the feasibility and accuracy of such a hybrid approach would be a valuable research endeavor in its own right.

At present, our dataset and modeling pipeline focus on real-world observed variations in HDR brachytherapy without explicitly embedding physics-based dose calculations. However, we appreciate your suggestion and believe that exploring a PhyML framework, could be a valuable direction for future work.

The limitations paragraph now reads as follows: “Finally, more computationally intensive approaches could be explored in future research. These include performing comprehensive hyperparameter optimization using dedicated libraries such as Optuna [69], evaluating additional ensemble-based methods like AdaBoost, or combining multiple algorithms. Other promising directions include the implementation of Multi-Feature Combined models as described by Yang et al. [70], as well as physics-informed ML [71] approaches that incorporate fundamental brachytherapy dose calculations to provide a physics-based foundation for data-driven model refinements.”

The following reference was added:

Karniadakis GE, Kevrekidis IG, Lu L, et al. Physics-informed machine learning. *Nat Rev Phys*. 2021;3(6):422-40. doi:10.1038/s42254-021-00314-5
