# Supplementary material for: Advancing patient care: Machine learning models for predicting grade 3+ toxicities in gynecologic cancer patients treated with HDR brachytherapy
Source: PLoS One. 2025 May 14;20(5):e0312208. doi: 10.1371/journal.pone.0312208 (PMC12077677; doi:10.1371/journal.pone.0312208)
Supplement: Attachment — Submitted filename: comments.docx [file pone.0312208.s010.docx]

The article titled "Advancing patient care: Machine learning models for predicting grade 3+ toxicities in

gynecologic cancer patients treated with HDR brachytherapy " was reviewed again.

This revision has significantly improved the manuscript. The authors have satisfactorily addressed my concerns.
